# Imunophenotypic Evaluation as a Tool for Monitoring Risks for Blood Malignancies in Gas Station Workers

**DOI:** 10.31557/APJCP.2019.20.7.2109

**Published:** 2019

**Authors:** Fabio Santiago, Simone Lima, Susani Antunes, Rafaele Tavares Silvestre, Luciano Rios Scherrer, Gilda Alves, Marilza de M Ribeiro- Carvalho, Maria Helena Ornellas

**Affiliations:** 1 *Laboratory of Circulating Markers, Department of Pathology and Laboratories, Faculty of Medical Sciences,*; 2 *Postgraduate in Medical Sciences (PGCM), Rio de Janeiro State University, Rio de Janeiro, *; 3 *Kennedy College, Belo Horizonte, Minas Gerais, Brazil. *

**Keywords:** Flow cytometry- lymphocyte phenotype- occupational exposure- human health risk assessment

## Abstract

**Background:**

Gas station workers are exposed to carcinogenic substances with impact on the hematologic and immune systems. The aim was to apply the immunophenotyping as a tool in the biological monitoring.

**Methods:**

This is a workplace-based case-control study with 49 workers and 26 controls. Medical interviews, hematological exams, and immunophenotyping analyses were performed. According to risk behavior (cleaning flannel and mistrust in the automatic fuel supply) the workers were divided into two groups: low risk (group 1) and high risk (group 2).

**Results:**

The results showed that CD16, HLA-DR, CD25, CD56+, CD16 CD56 low, and CD56 high expressions were higher in workers when compared to the control group (P =0.020, P =0.001, P =0.001; P =0.034, P=0.023, and P =0.008, respectively). The expressions of CD2, CD8, CD10, CD8low, and CD4/CD8 ratios were lower (P =0.016, P =0.001, P=0.001, P= 0.017, P = 0.0259, and P =0.029, respectively). Headache and paresthesia complaints were associated with workers when compared to the control group (OR = 4.091, 95% CI, 1.400 -11.951, P = 0.014; OR =12.12, 95% CI, 1.505 - 97.61, P =0.004). Using cleaning flannel and mistrust in the automatic fuel supply (risk behaviors) were associated with group 2 (OR = 9.71, 95% CI, 2.60-36.26, P = 0.005; OR = 18.18, 95% CI, 2.04-161.37, P = 0.004).

**Conclusions:**

The results strengthen the worker’s immunosuppression hypothesis, which may contribute to some disorders and the carcinogenesis process. The evaluation of the immune system by flow cytometry is a promising tool for monitoring blood malignancy risk in addition to regular classic hematological exams.

## Introduction

In Brazil, according to recent (2019) personal communication with a union representative, there are about 500,000 gas station workers, 20% of whom are females. They are chronically exposed to fuel vapors that contain established human carcinogens, such as BTEX (benzene, toluene, ethylbenzene, and xylenes) (Moura-Correa et al., 2014). According to IARC, benzene is considered the main carcinogenic agent (group 1 according to IARC), and the association with hematological diseases such as lymphoblastic leukemia (ALL), chronic lymphocytic leukemia (CLL), myelodysplastic syndromes (MDS), and myelogenous leukemia (AML), as well as different forms of non-Hodgkin’s lymphoma (NHL), has become clearer over the years (IARC, 2014; Stenehjen et al., 2015; Rushton et al., 2014; Schnatter et al., 2012). 

MDS and AML are the most frequent malignant neoplasms associated with BTEX exposure In addition there are evidences of the relationship between benzene and other types of hematological diseases such as other leukemias, lymphomas and multiple myeloma (De Roos et al., 2018). It is well known that severe bone marrow dysplasia is frequently accompanied by early alterations in the immune system and in peripheral cell subsets (Schnatter et al., 2012). The bone marrow injury by BTEX metabolites is a predisposing event in the pathogenesis of BTEX-induced hematopoietic disease (Smith, 2010). Thus, the early identification of this condition and cautioning the gas station workers about this risk may prevent the hematological disease progression. 

The immunophenotyping by flow cytometry is currently one of the fundamental pillars for the diagnosis and classification of lymphoproliferative and myeloid diseases (Craig and Foon, 2009). Immunologic dysregulation can be one cause of these diseases (Davidson-Moncada et al., 2018). The aim of the present study is to apply immunophenotyping as a tool in the biological monitoring of the gas station workers, helping in the diagnosis of benzene poisoning and in the prevention of hematological diseases.

## Materials and Methods

*Population study*

A total of 75 subjects were included in this study. The exposed group consisted of 49 workers (7 males and 42 females) recruited from 5 gas stations in the city of Rio de Janeiro. Gas stations were selected in a non-random way, seeking the ones with a larger number of women who could be more affected by BTEX exposure than men (Santiago et al., 2014; Santiago et al., 2017). In the control group a total of 26 unexposed subjects (9 males and 17 females) were selected among our university employees (professors and administrative staff) and students. This study was approved by the local ethics committee at the University of Rio de Janeiro State–UERJ (34310014.9.0000.5259/14)/ Brazil, and all subjects were informed about the potential benefits and risks of this study. 

Participation was voluntary and written informed consent was obtained from each participant in both groups. A physician questioned the members of the study population regarding their age, sex, race, life-style (smoking habits, alcohol and illicit drug consumption, etc.), and medical and work histories.

Classic hematological exam and biochemistry analysis

The hematological analysis consisted of hemogram and biochemistry analyzes. All blood tests were performed in the central laboratory of HUPE (Pedro Ernesto University Hospital), according to standard hematological methods.

*Immunophenotyping*

Two mL of EDTA-treated peripheral blood was prepared within 2 hours after collection using 18 antibodies (CD2, CD3, CD4, CD5, CD7, CD8, CD10, CD11c, CD16, CD19, CD22, CD20, CD25, CD27, CD45, CD56, and HLA-DR), according to standard procedures (Stelzer et al., 1997). The samples/antibodies were incubated for 30 min. The red cells were lysed with lysing solution (FACS Lysing Solution – Becton Dickinson). After being lysed, each sample was centrifuged twice, at 1000 rpm for 5 min. The cells were resuspended in 200µL of phosphate saline buffer (PBS - 0.01M, pH 7.4-7.6). The BD FACSCanto II cytometer was used and the data analyses were performed using the Infinicyt® software. The acquisition and analysis of immune-marked cells were standardized for 100,000 events per sample. Percentages were obtained for each lymphocyte sub-population and the absolute numbers of the lymphocyte sub-populations were also calculated. 

*Statistical analysis*

The workers were divided in two groups depending on BTEX exposure: group 1 with low risk and group 2 with high risk. The high risk of benzene poisoning was established based on the Brazilian Ministry of Health and the US Department of Labor guidelines for benzene poisoning diagnosis, using the following criteria:

(1) Number of leucocytes lower than 4,000 and/or neutrophils lower than 2,000; or anemia (low hematocrit: 40% for men and 36% for women); or thrombocytopenia (lower than 150,000);

(2) Report of four or more medical complaints (weight loss, weakness, dizziness, drowsiness, headache, irritability, nervousness, anxiety, insomnia, change of mood, depression, attention alteration, memory change, paresthesia, involuntary movements, tremor, and decreased muscle strength) related to benzene intoxication.

The groups were compared according to clinical characteristics, risk behaviors at work, classic hematological exams, and the immunophenotyping results. A non-parametric Mann-Whitney or Kruskall-Wallis test was used for comparison of the distributed variables between the groups. Also, the Pearson’s Chi-square or Fisher’s exact test was used to test the statistical significance of the association between clinical variables. The Predictive Analytics Software (PASW-Version 18) was used, and for all statistical tests P <0.05 was considered significant.

## Results

*Clinical findings and health report*

The gas station attendants routinely worked for 6 days per week, for 8 hours or more per day. The worker’s median age (group 1 and group 2) was 33.2 years, with 7.9 years of mean BTEX exposure time. The comparative analysis between the total of gas station workers and the control group for smoking, illicit drug consumption, alcohol consumption, and gender showed no significant P values (Table 1); thus, the comparative analysis of the data was performed without taking these variables into account. 

In the medical inquiries, anxiety (63.3%) was the most reported complaint, followed by headache (55.1%), irritability (46.9%), drowsiness (37.5%), paresthesia (32.7%), and others. The comparative analysis between the total of workers and the control group for headache and paresthesia showed significant P values for both variables associated with the workers, according to the Chi-Square test applied to Odds Ratio (OR) analysis (OR = 4.091, 95% CI, 1.400 -11.951, P = 0.014; OR =12.12, 95% CI, 1.505 - 97.61, P = 0.004, respectively) (Table 1). Note that no subjects reported carrying neoplasm, immunodeficiency, or self-immunity diseases.

For the comparative analysis of classic hematological exam results and exposure time between the workers group and the control group, Table 2 was created. An increased female CHGM, typical lymphocytes, and monocyte counts were observed in gas station workers (groups 1 and 2) related to the control group according to Kruskall-Wallis test (P =0.016, P =0.001, and P =0.003, respectively). In contrast, a decreased male hematocrit was found (P =0.015). Also, a higher gamma-glutamyltransferase (GGT) result was associated with group 1 compared to group 2, (average = 33.0 and P =0.012). Note that no more significant associations were found between groups 1 and 2 in classical hematological parameters independent of gender.

**Figure 1:**
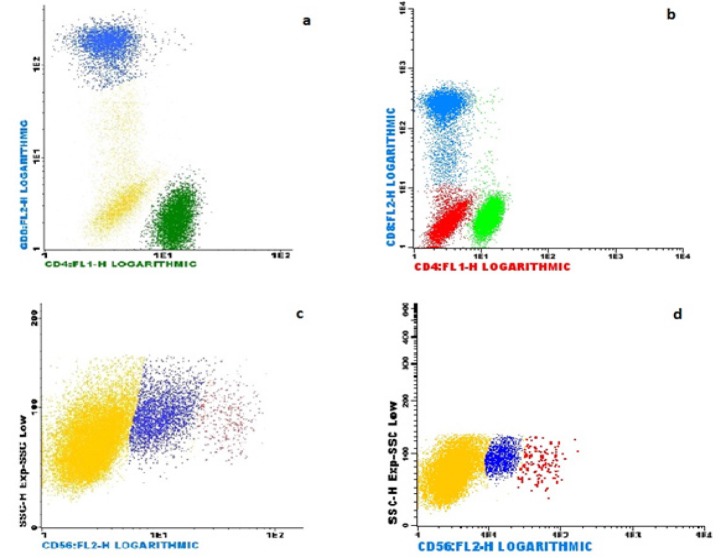
a, Shows the CD4 (35.54%) and CD8 (22.27%) cell expression of one female gas station worker, who has a low CD4 / CD8 ratio (1.59). In green the CD4 cells and in blue the CD 8 cells observed in the lymphocyte gate; b, Shows the CD4 (45.67%) and CD8 (24.57%) cell expression of one female control, who has 1.86 of CD4 / CD8 ratio. In green the CD4 cells and in blue the CD 8 cells observed in the lymphocyte gate; c, The flow cytometric analyses of NK cells (CD16 and CD56 positive) found in the lymphocyte gate of one female gas station worker. A high expression of NK cells with 17.14% of CD56 LOW (blue) and 2.46% of CD56 HIGH (red) was observed; d, The flow cytometric analyses of NK cells (CD16 and CD56 positive) found in the lymphocyte gate of one control female. Was observed a expression of NK cells with 2.38% of CD56 LOW (blue) and 0.23% of CD56 HIGH (red)

With regard to immunophenotyping data, we observed a higher expression of CD16, HLA-DR, CD25, CD56 + CD16, CD 56low, and CD 56high in lymphocytes in the workers (groups 1 and 2) compared to the control group (P =0.020, P =0.001, P =0.001; P =0.034, P =0.023, and P =0.008 respectively). In contrast, a lower expression of CD2, CD8, CD10, and CD8low was observed (P =0.016, P =0.002, P =0.001, and P =0.025, respectively). No more significant expression alterations were observed; however, the lower CD4 / CD8 ratio in the total of gas station workers compared to the control group should be highlighted (P =0.029) (Table 2). Figure 1 shows the immunophenotyping analysis of one female gas station worker who had alteration in the CD4/CD8 ratio and NK cell expression.

However, no significant association was found between groups 1 and 2 in monoclonal antibody results. To compare the variables of risk behaviors adopted by the workers between the groups, Chi-square tests were applied to Odds Ratio (OR) analysis of cleaning flannel use and mistrust in the automatic fuel supply (automatic pump device that stops the fuel supply when filling the car’s tank). The results revealed a significant effect of these risk behaviors on the workers in group 2 (OR = 9.71, 95% CI, 2.60-36.26, P = 0.005; OR = 18.18, 95% CI, 2.04-161.37, P = 0.004, respectively) (Table 3).

**Table 1:** Comparative Analysis of Demographic Data between Gas Station Workers and Control Group

Biometric data	Gas station Group	Control Group	P-value
Age (years)	33.3 (±9.9)	28 (±13.4)	0.1
Gender			0.129
Men	7 (14.3%)	8 (30.8%)	
Women	42 (85.7%)	18 (69.2%)	
Smokers			1
Yes	7 (14.3%)	3 (11.5%)	
No	42 (85.7%)	23 (88.5%)	
Illicit drug consumption			1
Yes	3 (6.1%)	2 (7.7%)	
No	46 (93.9%)	24 (92.3%)	
Drinking			0.62
Yes	30 (63.8%)	15 (57.7%)	
No	17 (36.2%)	11 (42.3%)	
Symptoms			
Weight Loss	10 (20.4%)	5 (19.2%)	1
Weakness	16 (32.7%)	3 (32.7%)	0.54
Dizziness	15 (32.6%)	4 (15.4%)	0.16
Drowsiness	18 (37.5%)	6 (24.0%))	0.3
Headache	27 (55.1%)	6 (23.1%)	**0.014**
Irritability / Nervousness	23 (46.9%)	7 (26.9%)	0.137
Anxiety	31 (63.3%)	11 (42.3%)	0.093
Insomnia	14 (28.6%)	3 (11.5%)	0.147
Change of mood / Depression	14 (28.6%)	5 (19.2%)	0.419
Attention Alteration	12 (25.0%)	5 (19.2%)	0,773
Memory change	13 (26.5%)	4 (14.4%)	0.387
Paresthesia	16 (32.7%)	1 (3.8%)	**0.004**
Involuntary movements	4 (8.2%)	1 (3.8%)	0.653
Tremors	8 (16.3%)	1 (3.8%)	0.15
Decreased muscle strength	6 (12.2%)	0 (0.0%)	0.09

**Table 2:** Comparative Analysis of Classic Hematological Exam and Immunophenotyping Results and Exposure Time between the Gas Station Workers and the Control Group

	Group 1	Group 2	Control group	p-value
Mean exposure time (year)	9.31 (±7.94)	7.17 (±6.27)	———	0.373
Blood test				
Platelets (mil/μL)	256.7 (±66)	286.4 (±71.3)	272.5 (±55.6)	0.282
Leukocytes (/μL	8425 (±2602)	6816 (±2094)	7532 (±1577)	0.144
Neutrophils (%)	56.29 (±8.60)	54.67 (±13.22)	60.53 (±9.12)	0.196
Eosinophils (%)	3.30 (±3.54)	2.66 (±2.12)	2.99 (±4.99)	0.171
Basophils (%)	0.49 (±0.53)	0.33 (±0.25)	0.43 (±0.53)	0.468
Granulocytes (%)	2.06 (±2.20)	1.50 (±1.21)	2.56 (±2.7)	0.064
Typical lymphocytes (%)	32.96 (±10.88)	32.40 (±12.60)	23.51 (±6.07)	**0.001**
Monocytes (%)	6.48 (±1.95)	6.00(±2.16)	4.73 (±1.37)	**0.003**
Gamma-GT (U/L)	33.04 (±20.38)	21.32 (±8.52)	21.20 (±12.06)	**0.012** *
Women				
Hemoglobin	13.10 (±1.24)	12.73 (±1.36)	12.99 (±0.65)	0.63
Hematocrit (%)	38.92 (±3.16)	38.23(±4.10)	39.7 (±1.55)	0.09
HGM (fl)	29.4 (±2.02)	29.08 (±1.80)	32.68 (±15.58)	0.768
CHGM (g/dL)	33.64 (±0.97)	33.29 (±0.94)	32.67 (±0.95)	**0.016**
VCM	87.33 (±4.59)	87.32 (±4.88)	87.67(±4.37)	0.981
Men				
Hemoglobin	14.82 (±0.45)	—————	14.76 (±2.03)	0.183
Hematocrit (%)	43.6 (±1.233)	—————	44.3 (±6.78)	**0.015**
HGM (fl)	29.4 (±2.02)	—————	32.68 (±15.58)	0.768
CHGM (g/dL)	34.01 (±0.88)	————	33.46(±1.21)	0.298
VCM	86.63 (±3.18)	—————	84.83 (±3.26)	0.418
CD2	76.16 (±19.80)	81.37 (±9.57)	85.84 (±3.71)	**0.016**
CD3	73.93 (±6.91)	76.43 (±9.63)	77.63 (±7.67)	0.344
CD4	46.61 (±10.21)	44.90 (±7.26)	42.06 (±12.33)	0.221
CD8	22.08 (±6.50)	24.06 (±6.76)	31.63 (±5.90)	**0.002**
CD 8 low	7.48 (±4.61)	6.91 (±2.95)	11.13 (±5.65)	**0.025**
CD4/CD8	2.27 (±0.80)	2.00 (±0.64)	3.97 (±6.46)	**0.029**
CD4 + CD8	0.92 (±0.83)	0.77 (±0.51)	0.66 (±0.48)	0.809
CD5	71.62 (±10.21)	70.02 (±9.61)	73.98 (±0.397)	0.397
CD7	73.95 (±13.49)	73.99 (±13.80)	75.91 (±10.18)	0.905
CD10	0.79 (±1.20)	1.45 (±4.16)	1.52 (±1.20)	**0.001**
CD11c	76.21 (±33.36)	78.69 (±27.10)	92.69 (±3.36)	0.811
CD16	10.31 (±6.15)	7.43 (±7.32)	12.09 (±28.58)	**0.02**
CD56	16.15 (±8.62)	14.09 (±8.69)	11.36 (±5.64)	0.178
CD56 low	14.66 (±8.24)	12.95 (±7.87)	7.85 (±6.45)	**0.023**
CD56 high	3.60 (±5.73)	2.69 (±4.22)	1.31 (±2.63)	**0.008**
CD16 + CD56	8.29 (±6.02)	6.13 (±5.13)	5.23 (±7.41)	**0.034**
NKT	3.75 (±4.57)	4.21 (±3.50)	6.20 (±6.20)	0.238
CD19	10.08 (±5.73)	10.51 (±4.81)	8.23 (±4.04)	0.285
CD20	9.66 (±5.39)	10.90 (±4.59)	9.37 (±3.45)	0.417
CD22	10.83 (±5.48)	11.40 (±4.28)	9.58 (±3.19)	0.28
CD25	8.91 (±8.71)	6.03 (±6.83)	1.66 (1.19)	**0.001**
CD27	52.77 (±10.39)	52.62 (±13.97)	72.16 (25.92)	0.059
HLA-DR	13.47 (±5.92)	16.38 (±7.92)	11.74 (4.29)	**0.04**

The results in bold are considered statistically significant , P <0.05.

* This was the only variable showing statistical difference between group 1 and group 2.

**Table 3:** Association between Risk Behavior of Gas Station Workers between Groups 1 and 2

Risk behavior at work	Group 1	Group 2	P-value	Odds Ratio	95% CI Odds Ratio
Uses the cleaning flannel	7 (29.2%)	20 (80%)	**0.001**	9.714	2.602 - 36.264
Smells the fuel cap before refueling	2 (9.5%)	6 (28.6%)	0.238	3.8	0.669 - 21,598
Holds the face close when fueling to the limit	11 (52.4%)	8 (38.1%)	0.536	0.559	0.164 - 1.911
Mistrust in the automatic fuel supply	1 (4.8%)	10 (47.6%)	**0.004**	18.182	2.048 - 161.37
Aspirates fuel with the mouth (hose)	6 (28.6%)	7 (33.3%)	1	1.25	0.337 - 4.639
Wet clothes with fuel	9 (42.9%)	10 (47.6%)	1	1.212	0.359 - 4.092

The results in bold are considered statistically significant, *P <0.05. *

## Discussion

In Brazil there is no self-service system of fuel and the female labor force at gas stations has been increasing exponentially in the last few years in our country. This is the reason for the interest focusing on the health of female gas station workers. A purposeful female predominance among young workers was observed, with 7.9 years of mean time exposure and a median age of 33.3 years.

According to the Brazilian Ministry of Health and the US Department of Labor, the diagnosis of benzene poisoning is clinically performed by an eminent physician and based on classic hematological exams (Brasil.Ministério da Saúde, 2016; Occupational Safety and Health Administration). With regard to symptoms, we observed a higher prevalence of many medical complaints, with statistical significance for headaches and paresthesia. Although Mitri et al., (2015) and Alves et al., (2017) reported similar symptoms in Brazilian gas station workers, in both studies there were no control groups to compare and assure the relevance of the reported data. In Alves et al., (2017) it was not clear whether workers were evaluated by a physician, as recommended by National and International Health Agencies.

It is worth noting that significant decreases in WBC, RBC, and platelet counts, and even the presence of isolated thrombocytopenia, have already been observed in human populations exposed to high levels of benzene in classic hematological exams (Abou-El Wafa et al., 2015; Smith, 2010). Abou-ElWafa et al.,(2015) reported a mean RBC count, hemoglobin level, and HCT significantly lower in gas station workers compared to the control group. In our study we found a range of cell alterations in classic hematological exams (female CHGM, typical lymphocytes, monocytes, male hematocrit), evidencing a non-specific cell effect and the clear involvement of the myeloid and lymphoid cell sets by BTEX exposure.

It is well known that all leukemias and MDS arise in the stem and progenitor cells of the bone marrow, which in chronic benzene exposure are clearly damaged by release of reactive oxygen species (ROS) produced by hydroquinone (benzene metabolite) oxidation reaction (Stenejjem et al., 2015). It is believed that the mechanisms of xylenes, toluene, and ethyl benzene-induced myelotoxicity are very similar to benzene, but this is still unclear (Smith, 2014). Also, it is well known that autoimmune alterations caused by initial bone marrow injury is an early or predisposing event in the pathogenesis of benzene-induced persistent hematopoietic disease.

With regard to immunophenotyping results, the increase in NK (CD56+CD16) cells recorded among BTEX workers was somewhat puzzling, especially considering the concomitant increase in CD 25 (IL-2) and DR+ cells, known as markers of activation of the immune system. Although the increase in NK cells would be inconsistent with immunosuppressive activity by BTEX, similar results were found by Bergamaschi et al., (1995), who studied the expression and activity of NK cells in workers exposed to styrene. Despite Bergamaschi et al.,’s findings (2015) of an increase in phenotypic NK cells (CD56+CD16), CD25, and DR+ cells, they proved a lower lytic activity by these NK cells in the exposed group. In agreement with this finding, Santiago et al., (2017) described a rare type of NK cells (NK bright) in peripheral blood of two Brazilian female gas station workers. This type of NK cells is known to have a low lytic activity with a high expression of cytokine. Note that CD25 (α chain of IL-2R) induces the activation and proliferation of not only NK cells, but also T cells, thymocytes, B cells, and macrophage populations. CD25 high expression in myelodysplastic syndrome may precede acute myeloid leukemia and chronic myeloid leukemia (Bergamaschi et al., 1995). Given the discrepancy with the above findings pointing to immunosuppression, the increase in CD25 and DR could be envisaged either as an unspecific response or as a compensatory mechanism in response to the low cell proliferations caused by BTEX exposure. 

In agreement with the workers’ immunosuppression hypothesis, a low CD4/CD8 ratio was observed compared to the control group. This low ratio indicates a disproportion of T helper (CD4) and T suppressor (CD8) lymphocytes, as observed in patients with severe immunosuppression, such as in AIDS patients. It should also be noted that in MDS patients this ratio is frequently inverted, and there is an increase in cytotoxic CD8+ T cells (CTL) as well (Nakase et al., 2017).

It should be noted that none of the classic hematological exam and immunophenotyping results was associated with either group (1 or 2) of workers. Thus, we questioned the guidelines adopted for benzene poisoning diagnosis based only on the symptomatology and classic hematological exams, since innumerable alterations were found when comparing the data between the control group and the total of gas station workers. However, it is worth noting that the GGT results were associated with group 1, with less medical complaints. Liu et al., (2009) suggest that GGT within its reference range predicts several clinical outcomes as a sensitive marker of oxidative stress in humans and can mark exposure to various environmental pollutants. Epidemiological findings about GGT serum imply the possibility of harmful effects of various environmental pollutants at background levels currently regarded as safe.

Note that the use of cleaning flannel and the mistrust in the automatic fuel supply by the workers were associated with group 2, with the highest medical complaints. The cleaning flannel wet with fuel remains most of the time on the attendants’ shoulder, and the workers who mistrust the automatic fuel supply hold their face close to the fuel tank to hear or to see the gasoline rising when fueling the car, thereby increasing the BTEX exposure. Thus, the workers’ orientation regarding the non-adoption of these behaviors would reduce their symptoms and possibly the carcinogenic effects. 

In conclusion in agreement with the immunosuppression hypothesis of these workers, a range of changes were found in CD16, HLA-DR, CD25, CD56+CD16, CD56 high, CD56 low, CD2, CD8, CD8 low, and CD10 expression, as well as a low CD4/CD8 ratio compared with the control group. Not only were risk behaviors at work (the use of cleaning flannel and mistrust in the automatic fuel supply) identified and associated with high risk of benzene poisoning, but also the results indicated that workers exposed to BTEX have their immune system impaired, which may contribute to some disorders and the carcinogenesis process. The evaluation of the immune system by flow cytometry is a promising tool in the blood monitoring of these workers in addition to normal classic hematological exams. The BTEX poisoning diagnosis and the carcinogenesis process identification are impaired when we take into account only the medical complaints/symptomatology and the classic hematological exam results. 

Unfortunately, the toxicological assessment studies of gas station workers are still insufficient to fulfill the demand of these workers and the identification of the real risks. Further studies are needed, especially those related to the biological monitoring and the immune system of gas station workers. 

## Data Availability

All the data supporting our findings are contained within the manuscript.
